# Incidence of Dupuytren’s disease following hand trauma: a systematic review

**DOI:** 10.1177/17531934251360545

**Published:** 2025-08-01

**Authors:** Jennifer Novo, David Gao, Ishith Seth, Warren M Rozen

**Affiliations:** 1 School of Medicine, University of Notre Dame Sydney, Darlinghurst, NSW 2010, Australia; 2 School of Medicine and Dentistry, Griffith University, Gold Coast, QLD 4222, Australia; 3 Department of Plastic and Reconstructive Surgery, Peninsula Health, Frankston, VIC 3199, Australia; 4 Peninsula Clinical School, Faculty of Medicine, Monash University, Melbourne, VIC 3004, Australia

**Keywords:** Dupuytren’s disease, fibroproliferative condition, hand trauma, incidence, palmar fascia, surgical trauma

## Abstract

Trauma, particularly surgical trauma, has been suggested as a potential trigger for Dupuytren’s disease (DD). This systematic review examined the prevalence of DD after surgical and non-surgical hand trauma by conducting thorough searches of the PubMed, Embase, Cochrane and Scopus databases. Qualitative methods were used to synthesise the data and summarize the findings that were unsuitable for meta-analysis. The findings revealed an increased risk of DD associated with exposure to hand-transmitted vibration, sports-related trauma and distal radial fractures. Surgical procedures such as trigger finger release were also found to be associated with an increased risk, particularly in individuals who are predisposed to the condition. Key risk factors included age, male sex, diabetes and smoking. Hand trauma, particularly surgical trauma and repetitive mechanical stress, is associated with the onset of DD. These findings highlight the need to consider the risk of developing DD in patients undergoing surgery or sustaining an injury. Further research is needed to develop preventive strategies for at-risk populations.

## Introduction

Dupuytren’s disease (DD) is a progressive fibroproliferative disorder of the palmar fascia, leading to flexion contractures that impair hand function (Chung, 2014; Seth et al., 2025). It predominantly affects Caucasian males of Northern European descent, with an estimated global prevalence of 8.2% (Salari et al., 2020). While the aetiology is complex, genetic predisposition plays a primary role, with emerging evidence implicating mechanical stress and trauma as potential contributing factors (Tripoli et al., 2016; van den Berge et al., 2023).

At the cellular level, DD is characterized by excessive extracellular matrix (ECM) deposition, driven by aberrant myofibroblast activity (Chung, 2014; Tripoli et al., 2016). While transforming growth factor-beta (TGF-*β*) is a key regulator of myofibroblast differentiation, recent evidence suggests that tumour necrosis factor (TNF) also drives persistent fibrosis by dysregulating ECM turnover (Forsman, 2016; Layton and Nanchahal, 2019; Verjee et al., 2013). Rather than initiating the disease, myofibroblasts contribute to a self-sustaining fibrotic process in a predisposed ECM environment, leading to fascia thickening, nodule formation and pathological cords that cause contractures (Layton et al., 2023; Tripoli et al., 2016). Repetitive strain or trauma to the palmar fascia may similarly disrupt normal tissue homeostasis, triggering an exaggerated fibrotic response that promotes disease progression (Layton et al., 2023). In DD, aberrant TNF and TGF-*β* signalling pathways contribute to persistent fibrosis, providing a potential explanation for the association between mechanical stress and disease onset (Layton et al., 2023; Verjee et al., 2013).

Several hypotheses have been proposed to explain how trauma contributes to DD. One theory suggests that the trauma and subsequent wound healing processes associated with an injury to the palmar fascia may induce fibroblastic activity in susceptible individuals, thereby promoting the development of DD (Evans et al., 2002). Another hypothesis is that surgical or mechanical trauma may unmask pre-existing but subclinical DD through changes in local tissue dynamics and increased mechanical stress on the palmar fascia (Samulėnas et al., 2020). Additionally, repetitive strain and vibration exposure, common in manual labourers and those engaged in physically demanding activities, have been associated with increased DD risk (Descatha et al., 2011).

Surgical trauma, particularly from procedures such as carpal tunnel release and trigger finger surgery, has been associated with postoperative DD onset (Maasarani et al., 2023; Samulėnas et al., 2020; Wroblewski, 1973). It is hypothesized that surgical manipulation of the palmar fascia may induce localized inflammation and tissue remodelling, exacerbating fibrotic changes in genetically predisposed individuals (McFarlane, 1991). While some studies have explored these associations, the broader question of whether different types of trauma, surgical or otherwise, can trigger or accelerate DD progression remains underexplored. Understanding these relationships is crucial for identifying at-risk populations and informing preventive and post-surgical management strategies.

This review aims to critically examine the current literature to determine whether trauma, in its various forms, contributes to the development of DD and to explore the underlying mechanisms of this association. By synthesizing data from diverse studies, this review seeks to identify patterns, address inconsistencies and highlight gaps in the existing research. These insights may help inform clinical decision-making, improve preventive strategies and guide future investigations to understand trauma-related DD pathogenesis better.

## Methods

We performed a systematic review of the existing literature on the new onset of DD following surgical and non-surgical trauma to the hand. The review protocol was registered with PROSPERO (ID: CRD42024575145).

Two authors (JN and DG) systematically searched PubMed, Embase, Cochrane, and Scopus, from 1901 to August 2024. The search used Medical Subject Headings (MeSH) terms and various free-text keywords such as ‘Dupuytren’s disease’, ‘contracture’, ‘surgery’, ‘new onset’, ‘incidence’, ‘palmar fascia’, ‘trigger finger release’, ‘de Quervain’s release’, ‘carpal tunnel release’, ‘wrist arthroscopy’ and ‘surgical trauma’. Boolean operators (AND, OR) were used to ensure broad topic coverage and refine the search results. The full electronic search strategies for all databases, including complete MeSH terms and Boolean logic, are provided in Online File S1. Only English-language articles were included. After excluding duplicates and irrelevant articles, the initial search was supplemented by manually reviewing the reference lists of identified studies to capture additional relevant articles not retrieved in the database searches.

Covidence (Veritas Health Innovation, 2024), an online systematic review tool, was used to screen studies, extract data, and conduct quality assessments. Two independent reviewers used Covidence to screen titles, abstracts, and subsequent full texts to ensure a consistent and transparent screening process. A third reviewer (IS) resolved conflicts during the screening phase.

### Inclusion and exclusion criteria

We included studies that investigated patients 18 years and older with no prior diagnosis of DD, focusing specifically on the incidence of DD following any hand trauma (surgical and non-surgical). Only peer-reviewed original research articles were considered, including randomized controlled trials, cohort, case–control and cross-sectional studies. Additionally, studies had to provide detailed information on patient populations, methods, and outcomes.

Studies were excluded if they involved paediatric patients (under 18 years) or animals. Non-original research, such as reviews, case reports, case series, editorials, letters to the editor or expert opinions, was also excluded. Studies not involving hand trauma (e.g. trauma to other body parts) or not reporting data on the incidence or prevalence of DD were excluded. Conference abstracts and unpublished studies were also excluded.

### Data extraction and synthesis

Data extraction was performed using Covidence by two independent reviewers (JN and DG) to ensure consistency and accuracy across all studies, with any conflicts resolved by a third reviewer (IS). Extracted data included the author’s name, year of publication, study design, patient demographics, sample size, type of hand trauma or surgery, findings, identified risk factors associated with DD and key conclusions. Data synthesis involved qualitative methods to assess the incidence of DD. A narrative synthesis summarized findings from studies unsuitable for meta-analysis owing to heterogeneity in study design, population or outcome measures. Discrepancies between reviewers were resolved through discussion or consulting a third reviewer (IS).

### Quality assessment

Tools were used for quality assessment depending on the study type. The Newcastle–Ottawa Scale (NOS) was used for cohort and case–control studies, evaluating study group selection, comparability, and outcome ascertainment. The Joanna Briggs Institute (JBI) Critical Appraisal Tool for Analytical Cross-Sectional Studies was used for cross-sectional studies. No randomized controlled trials were included in this review. The JBI checklist comprises eight questions that address sampling, data collection and reporting reliability. Each question allowed responses of either Yes, No, Unclear or Not Applicable, providing a structured framework for evaluating each the validity and applicability of each study. Two reviewers (JN and DG) assessed each study independently, resolving any disagreements through discussion or consultation with a third reviewer (IS).

## Results

The search process identified 1532 studies in the databases. After removing duplicates and ineligible studies, and screening the titles and abstracts, 61 studies were selected for a full-text review. Of these, 22 studies met the inclusion criteria and were included in this review (Online Fig. S1).

### Study characteristics

The 22 included studies were published between 1971 and 2024 and covered a variety of countries, study designs and sample sizes. Most were cross-sectional or cohort studies, with one case–control study. Sample sizes ranged from fewer than 100 participants to large-scale population studies involving more than 21,000 participants. Eleven studies relied on self-reported data collection methods, while the remainder were based on clinical diagnosis. The studies investigated various types of hand trauma, including surgical procedures, exposure to vibration, sports-related injuries and fractures. Key study characteristics are summarized in Table 1.

**Table 1.** Summary of characteristics of included studies (year, author, design, country, sample size, trauma type, mean age in years, sex and key findings)

Author and year	Design	Country	Sample size	Trauma type(s)	Mean age (years)	Sex	Key findings
Zachariae (1971)	Cross-sectional study	Denmark	860	Acute non-surgical and manual labour	n/a	Both	Trauma and manual work are not key factors for Dupuytren’s
Bell (1977)	Retrospective cohort study	UK	53	Acute non-surgical, manual labour	n/a	Men	Manual labour accelerates the onset of Dupuytren’s disease in predisposed individuals
Mikkelsen (1978)	Cross-sectional study	Norway	15,950	Acute non-surgical, manual labour	n/a	Both	Higher prevalence of Dupuytren’s disease in manual labourers. Men more affected
Bennett (1982)	Cross-sectional study	UK	216	Manual labour	n/a	Men	Higher Dupuytren’s incidence in workers in the bagging and packing industry. Most cases are mild
Stewart and Burke (1985)	Prospective cohort study	UK	235	Colle’s fracture	60.2	Predominantly women	11% Dupuytren’s incidence post-Colles’ fracture. Older age is a factor
Kelly et al. (1992)	Observational cohort study	UK	235	Acute non-surgical	67.3	Predominantly women	9.3% Dupuytren’s incidence after Colles’ fracture. Nodules and skin pits are common
Dasgupta and Harrison (1996)	Cross-sectional study	India	66	Vibration exposure	n/a	Men	No link between vibration and Dupuytren’s. Neurological symptoms are more common in drillers
Livingstone and Field (1999)	Prospective observational study	UK	100	Acute non-surgical	70	Predominantly women	Dupuytren’s disease is more prevalent in patients with algodystrophy following a radius fracture
Logan et al. (2005)	Cross-sectional study	UK	545	Rock climbing	54	Men	Rock climbers have 19.5% Dupuytren’s prevalence. Earlier onset seen
Burke et al. (2007)	Cross-sectional study	UK	97,537	Vibration exposure	n/a	Men	Age, smoking, alcohol, and diabetes linked to Dupuytren’s. No link with vibration exposure
Abe et al. (2007)	Retrospective cohort study	Japan	133	Acute surgical, non-surgical	56	Both	13 patients (9.8%) developed DD following trauma; average onset was 3.6 months
Lucas et al. (2008)	Cross-sectional study	France	2,406	Vibration exposure and manual labour	50.7	Men	Manual work and vibration are linked to Dupuytren’s. The trauma link reduced after adjustment
Descatha et al. (2012)	Cross-sectional study	France	2,161	Vibration exposure	47.1	Men	Manual work and vibration are linked to Dupuytren’s. Dose–response observed
Palmer et al. (2014)	Cross-sectional study	United Kingdom	4,969	Vibration exposure, manual labour	n/a	Men	Hand-transmitted vibration exposure triples the risk of Dupuytren’s disease in men. Manual workers show a higher risk
Descatha et al. (2014)	Prospective cohort study	France	13,587	Vibration exposure	66.5	Both	Dupuytren’s is linked to age, diabetes and long-term vibration exposure in men
Broekstra et al. (2018)	Cross-sectional study	Netherlands	325	Field hockey	n/a	Men	Field hockey is linked to Dupuytren’s. No dose-response found
Haines et al. (2017)	Case–control study	Canada	358	Acute non-surgical, chronic occupational exposure	54.2	Both	Repetitive handwork increases Dupuytren’s risk. No link found with urgent hand injuries
Samulėnas et al. (2020)	Retrospective Observational Study	Lithuania	132	Acute surgical and non-surgical	60	Both	22% injury-induced Dupuytren’s. Slower progression and milder contractures
Van den Berge et al. (2021)	Prospective cohort study	The Netherlands	763	Manual labour	n/a	Both	Hand injury increases Dupuytren’s risk. Manual labour is not significant
Maasarani et al. (2023)	Retrospective cohort study	USA	85,944	Acute surgical (trigger finger release)	n/a	Both	Trigger finger surgery increases the risk of Dupuytren’s. Faster onset post-surgery
Van den Berge et al. (2023)	Population-based cohort study	UK	196,265	Chronic occupational exposure	53	Both	Manual labour shows a dose–response link to Dupuytren’s. Supports work-related classification
Zimmerman et al. (2024)	Retrospective cohort study	Sweden	14,342	Vibration exposure	57	Both	No link between vibration and Dupuytren’s. Genetics and diabetes are the likely causes

### Quality assessment

In the context of cohort, cross-sectional and case–control studies, the majority demonstrated a low risk of bias when evaluated using the JBI checklist and the NOS (Online Tables S1–S3). Cross-sectional studies performed particularly well, with most achieving 7 out of 8 points on the JBI checklist, reflecting reliable sampling and data collection. Cohort studies scored between 6 and 8 out of 9 on the NOS, indicating strong methodological rigour in areas such as study design, comparability and outcome assessment. The single case study scored 7 out of 9, indicating good comparability and exposure assessment.

The most notable source of bias was the reliance on self-reported data, which introduced potential recall bias, affecting exposure ascertainment. This was particularly evident in studies such as those by Lucas et al. (2008) and Palmer et al. (2014), where reliance on participant recollection limited the precision of reported outcomes. Some cohort studies, including Maasarani et al. (2023), had shorter follow-up periods, which limited their ability to assess delayed onset of DD. Cross-sectional studies, such as Zachariae (1971), often lacked clarity in their sampling methods, which introduced potential selection bias, which is more commonly observed in older studies. Other minor sources of bias included variability in diagnostic criteria for DD and inconsistent reporting of DD severity or progression across studies (Abe et al., 2007; Bennett, 1982; Broekstra et al., 2018; Descatha et al., 2011). Despite these limitations, these studies were included to ensure the inclusion of a broad range of studies examining trauma-related DD.

### Incidence of DD following trauma

#### Non-surgical trauma

The incidence of DD varied across the studies, depending on the type of trauma. Among non-surgical trauma, studies examining vibration exposure consistently reported a significantly increased risk of DD. Palmer et al. (2014) observed a threefold increase in risk among workers exposed to hand-transmitted vibration, with a prevalence ratio of 3.15 (95% CI: 1.96 to 5.05). Similarly, Descatha et al. (2012) reported an odds ratio of 10.8 (95% CI: 3.4 to 34.6) in workers with additional risk factors such as diabetes. In contrast, however, Zimmerman et al. (2024) reported no association between vibration exposure and DD, and suggested that individual susceptibility factors, such as genetic predisposition or variations in the intensity and duration of exposure, may moderate the risk.

In populations exposed to repetitive hand strain, the prevalence of DD was notably high. Logan et al. (2005) reported a 20% prevalence of DD in rock climbers, with a significant correlation to climbing intensity. Similarly, Broekstra et al. (2018) observed a higher incidence of DD (15%) in field hockey players. Across studies, the prevalence of DD in individuals exposed to repetitive strain ranged from 15 to 19.5%. Both Broekstra et al. (2018) and Logan et al. (2005) highlighted sports-related trauma as a factor associated with onset of the disease.

#### Acute trauma

Wide variations were reported in DD incidence following fractures. Livingstone and Field (1999) found a 39% incidence within 18 months of distal radial fractures, while Stewart and Burke (1985) reported a lower incidence of 11% following Colles’ fracture. These discrepancies are probably due to the differences in follow-up periods and diagnostic criteria. Furthermore, Abe et al. (2007) reported that five out of 133 patients developed DD following acute non-surgical trauma, with an average onset time of 3.6 months after the injury.

#### Surgical trauma

Surgical procedures such as trigger finger release and carpal tunnel release were associated with an increased risk of developing DD in predisposed individuals. Samulėnas et al. (2020) reported an incidence of 2.3% postoperatively, while Maasarani et al. (2023) found that patients who underwent trigger release had a significantly higher risk of developing DD (OR: 1.25) compared with those who underwent conservative management such as steroid injections. They were also significantly more likely to require fasciectomy (OR: 1.776; 95% CI: 1.14 to 2.76).Abe et al. (2007) similarly observed that eight out of 133 patients developed DD following surgical trauma, most commonly after carpal tunnel release or flexor tendon repair, with an average onset of 3.6 months post-procedure.

#### Risk factors

Several studies identified common risk factors associated with the development of DD post-trauma, including age, male sex, smoking and diabetes (Burke et al., 2007; Descatha et al., 2012; Haines et al., 2017). The definition of ‘heavy smoking’ varied, with Burke et al. (2007) defining it as consuming 20 or more cigarettes per day, and (Descatha et al., 2014) categorizing smokers as consuming either less than or more than one pack per day. Other studies, such as Haines et al. (2017) and Broekstra et al. (2018), referenced smoking qualitatively without specifying thresholds. Haines et al. (2017) found that heavy smoking and repetitive handwork increased the risk of DD by 1.03 per year of exposure (*p* = 0.04). Descatha et al. (2014) found a strong association between alcohol intake, occupational exposure and DD in men. This suggests that lifestyle factors may amplify the incidence of DD in workers who use vibrating tools. Furthermore, Broekstra et al. (2018) confirmed these findings in a study of field hockey players, revealing that a history of heavy smoking or alcohol consumption was associated with a higher incidence of DD.

## Discussion

This systematic review found that both surgical and non-surgical hand trauma may be associated with the development of DD, particularly in genetically predisposed individuals (Haines et al., 2017; Kelly et al., 1992; Maasarani et al., 2023). Repetitive mechanical stress and vibration exposure emerged as the most consistently linked factors, while evidence for surgical trauma was more variable (Palmer et al., 2014; van den Berge et al., 2023). While several studies identified strong associations, others found no significant link, suggesting that trauma alone may not be sufficient to trigger DD in the absence of other modifying factors (Dasgupta and Harrison, 1996; Zimmerman et al., 2024).

Emerging mechanistic insights suggest that trauma-related inflammation plays a key role in disease initiation. Injuries such as fractures, lacerations and repetitive strain have been shown to trigger fibroblast activation and fibrotic cascades (Abe et al., 2007; Balakrishnan et al., 2008; Findlay and Tahmassebi, 2014). The persistent infiltration of immune cells, particularly macrophages and T-helper cells, into palmar tissue may drive chronic inflammation through cytokines such as TNF, IL-6 and IL-8, promoting fibroblast-to-myofibroblast differentiation (Layton et al., 2023). Rather than resolving through normal wound healing, this prolonged inflammatory signalling appears to facilitate fibrosis in susceptible individuals.

In addition to immune activation, vibration-induced cellular stress may upregulate profibrotic pathways like TGF-*β*, accelerating myofibroblast contraction and collagen deposition (Balakrishnan et al., 2008; Cevik et al., 2025; Forsman, 2016; Layton and Nanchahal, 2019). Chronic mechanical loading may also compromise microvascular integrity and induce local hypoxia, both of which contribute to tissue remodelling and nodule formation (Descatha et al., 2011; Gerger et al., 2023; Liss and Stock, 1996). Clinical observations further support this mechanistic link, as many DD patients retrospectively report preceding hand trauma (Rayan and Moore, 2005). These findings may ultimately inform the development of targeted therapies to halt fibrotic progression at a molecular level.

Trauma-related risk appears to vary by context. While some studies demonstrated a link between repetitive strain or vibration exposure and DD onset (Haines et al., 2017; Palmer et al., 2014), others found no such association (Zimmerman et al., 2024). Nonetheless, prior systematic reviews support the hypothesis that trauma may influence DD onset in specific populations (Gerger et al., 2023; Mathieu et al., 2020), suggesting that genetic predisposition or exposure intensity may be critical modifiers. Similarly, surgical trauma was associated with DD onset in some studies, particularly following carpal tunnel release or trigger finger surgery (Abe et al., 2007; Maasarani et al., 2023; Samulėnas et al., 2020). This may reflect iatrogenic stress to the palmar fascia, highlighting the importance of preoperative risk stratification and surgical refinement in vulnerable individuals.

Environmental risk factors also intersect with occupational exposure. Manual labour involving repetitive hand use or vibration has been linked to increased DD risk across multiple studies (Bell, 1977; Layton and Nanchahal, 2019; Palmer et al., 2014). The proposed mechanism involves chronic mechanical strain and microvascular compromise, leading to myofibroblast activation (Findlay and Tahmassebi, 2014; Layton and Nanchahal, 2019; van den Berge et al., 2023). These insights support practical interventions, such as ergonomic adjustments and vibration reduction strategies, to mitigate cumulative trauma (Mathieu et al., 2020).

Sport-related trauma has also been implicated, particularly in activities requiring high-frequency gripping or impact. Elevated rates of DD have been reported in specific athlete groups, including rock climbers and field hockey players (Broekstra et al., 2018; Logan et al., 2005). Repetitive hand stress in these contexts may activate similar fibrotic pathways (Bennett, 1982; Broekstra et al., 2018; Layton and Nanchahal, 2019; Logan et al., 2005; Mikkelsen, 1978; van den Berge et al., 2021). For athletes with underlying genetic or occupational risk, trauma exposure may accelerate disease onset (McFarlane, 1991). Clinicians and coaches should be aware of this risk, and early recognition in high-demand athletes may help guide monitoring and preventive strategies.

Demographic factors such as age and sex also appear to influence susceptibility. Older males engaged in high-strain occupations or contact sports have been consistently identified as higher risk groups (Burke et al., 2007; Haines et al., 2017). This aligns with the known epidemiology of DD, which disproportionately affects older Caucasian males of Northern European descent (McFarlane, 1991; Rayan and Moore, 2005). Age-related declines in regenerative capacity may further exacerbate trauma-induced tissue changes (Lucas et al., 2008; McFarlane, 1991). These findings underscore the need for targeted prevention strategies that focus on older males in high-exposure environments.

Clinically, increased awareness of trauma-related DD risk is essential. Surgeons should incorporate trauma history and occupational exposure into preoperative assessment protocols to better identify at-risk patients. For those undergoing procedures such as carpal tunnel release, postoperative monitoring may help detect early fibrotic changes (Bennett, 1982). Similarly, early recognition among athletes and workers could facilitate timely interventions to preserve function and delay progression.

Preventive efforts may include workplace ergonomic modifications, vibration reduction and targeted education for individuals in high-risk occupations or sports (Descatha et al., 2011; Mikkelsen, 1978). While direct evidence linking ergonomic interventions to reduced DD incidence is limited, similar approaches have proven effective in lowering rates of other musculoskeletal disorders (Bernard and Putz-Anderson, 1997). Hand therapists can support these interventions by educating patients on tool ergonomics, grip modification and load distribution, particularly in manual labour environments. In athletic settings, individuals with predisposing factors may also benefit from protective strategies, such as adjusting equipment or making structured training modifications. These proactive efforts across occupational and recreational domains may help mitigate the long-term risk of fibrosis in susceptible populations.

This review has several limitations. The absence of a meta-analysis limited the ability to quantify effect sizes across studies, reducing statistical power. The substantial heterogeneity among the included studies, in terms of study design, diagnostic criteria, populations and outcome measures, made direct comparisons challenging and precluded a meta-analysis. Additionally, many studies had methodological shortcomings – small sample sizes and short follow-up periods – which restricted the applicability of findings and hampered the ability to establish clear temporal relationships (Abe et al., 2007; Stewart and Burke, 1985). Eleven studies relied at least partially on self-reported data, introducing potential recall bias. Outcome assessment methods also varied widely, which may have contributed to inconsistent results. The predominance of cross-sectional studies further limited the ability to make causal inferences. Finally, the exclusion of non-English studies may have led to the omission of valuable data.

Future research should prioritize longitudinal studies that track DD onset following different types of hand trauma, using standardized diagnostic criteria to improve comparability (Abe et al., 2007). Mechanistic studies exploring pathways such as TGF-*β* signalling may clarify the link between trauma and fibrotic progression (Findlay and Tahmassebi, 2014). When feasible, meta-analyses could enhance statistical power and causal inference. Clarifying the role of trauma in DD pathogenesis may support the development of targeted prevention and management strategies in at-risk populations.

## Supplemental Material

sj-xlsx-1-jhs-10.1177_17531934251360545 - Supplemental material for Incidence of Dupuytren’s disease following hand trauma: a systematic review Supplemental material, sj-xlsx-1-jhs-10.1177_17531934251360545 for Incidence of Dupuytren’s disease following hand trauma: a systematic review by Jennifer Novo, David Gao, Ishith Seth and Warren M Rozen in Journal of Hand Surgery (European Volume)

sj-pdf-2-jhs-10.1177_17531934251360545 - Supplemental material for Incidence of Dupuytren’s disease following hand trauma: a systematic review Supplemental material, sj-pdf-2-jhs-10.1177_17531934251360545 for Incidence of Dupuytren’s disease following hand trauma: a systematic review by Jennifer Novo, David Gao, Ishith Seth and Warren M Rozen in Journal of Hand Surgery (European Volume)

sj-pdf-3-jhs-10.1177_17531934251360545 - Supplemental material for Incidence of Dupuytren’s disease following hand trauma: a systematic review Supplemental material, sj-pdf-3-jhs-10.1177_17531934251360545 for Incidence of Dupuytren’s disease following hand trauma: a systematic review by Jennifer Novo, David Gao, Ishith Seth and Warren M Rozen in Journal of Hand Surgery (European Volume)

sj-pdf-4-jhs-10.1177_17531934251360545 - Supplemental material for Incidence of Dupuytren’s disease following hand trauma: a systematic review Supplemental material, sj-pdf-4-jhs-10.1177_17531934251360545 for Incidence of Dupuytren’s disease following hand trauma: a systematic review by Jennifer Novo, David Gao, Ishith Seth and Warren M Rozen in Journal of Hand Surgery (European Volume)

sj-pdf-5-jhs-10.1177_17531934251360545 - Supplemental material for Incidence of Dupuytren’s disease following hand trauma: a systematic review Supplemental material, sj-pdf-5-jhs-10.1177_17531934251360545 for Incidence of Dupuytren’s disease following hand trauma: a systematic review by Jennifer Novo, David Gao, Ishith Seth and Warren M Rozen in Journal of Hand Surgery (European Volume)
