# Supplementary material for: Incidence of Dupuytren’s disease following hand trauma: a systematic review
Source: J Hand Surg Eur Vol. 2025 Aug 1;51(1):6–13. doi: 10.1177/17531934251360545 (PMC12705875; doi:10.1177/17531934251360545)
Supplement: sj-pdf-2-jhs-10.1177_17531934251360545 - Supplemental material for Incidence of Dupuytren’s disease following hand trauma: a systematic review [file sj-pdf-2-jhs-10.1177_17531934251360545.pdf]

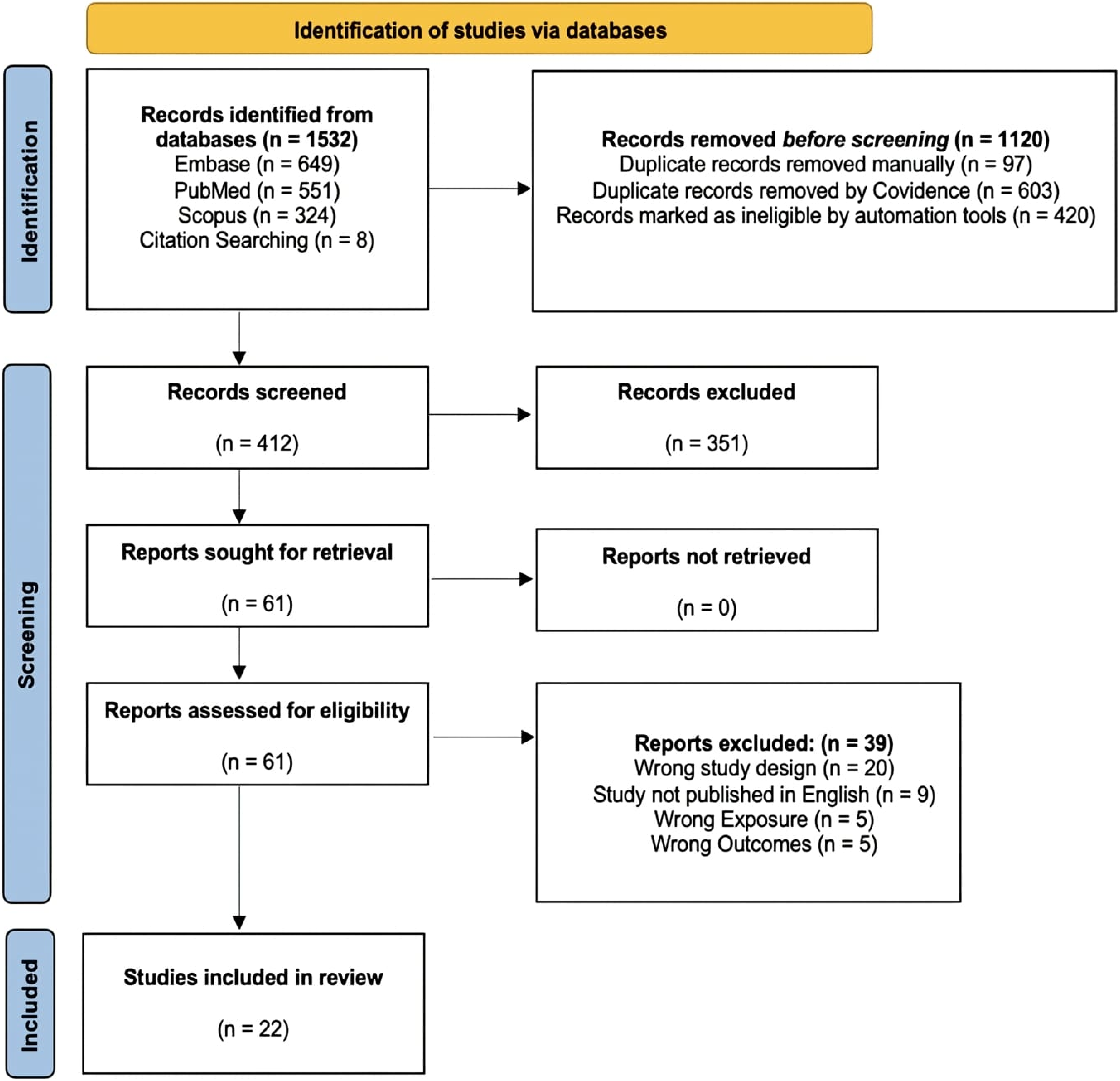

Identification of studies via databases
Identification
Records identified from databases (n = 1532)
Embase (n = 649)
PubMed (n = 551)
Scopus (n = 324)
Citation Searching (n = 8)
Records removed *before screening* (n = 1120)
Duplicate records removed manually (n = 97)
Duplicate records removed by Covidence (n = 603)
Records marked as ineligible by automation tools (n = 420)
Screening
Records screened
(n = 412)
Records excluded
(n = 351)
Reports sought for retrieval
(n = 61)
Reports not retrieved
(n = 0)
Reports assessed for eligibility
(n = 61)
Reports excluded: (n = 39)
Wrong study design (n = 20)
Study not published in English (n = 9)
Wrong Exposure (n = 5)
Wrong Outcomes (n = 5)
Included
Studies included in review
(n = 22)
