# Supplementary material for: Incidence of Dupuytren’s disease following hand trauma: a systematic review
Source: J Hand Surg Eur Vol. 2025 Aug 1;51(1):6–13. doi: 10.1177/17531934251360545 (PMC12705875; doi:10.1177/17531934251360545)
Supplement: sj-pdf-3-jhs-10.1177_17531934251360545 - Supplemental material for Incidence of Dupuytren’s disease following hand trauma: a systematic review [file sj-pdf-3-jhs-10.1177_17531934251360545.pdf]

Online Table S1. Quality assessment of included studies using the Joanna Briggs Institute (JBI) critical appraisal checklist for analytical cross-sectional studies

Author & year	Q1	Q2	Q3	Q4	Q5	Q6	Q7	Q8
Zachariae (1971)	N	Y	N	Y	N	Y	N	N
Mikkelsen (1978)	N	Y	Y	Y	N	NA	U	U
Bennett (1982)	N	Y	N	U	Y	N	Y	Y
Dasgupta et al. (1996)	U	Y	N	N	Y	Y	Y	Y
Logan et al. (2005)	U	Y	N	N	Y	N	U	Y
Burke et al. (2007)	U	N	N	N	Y	Y	Y	Y
Lucas et al. (2008)	N	Y	Y	Y	Y	Y	Y	Y
Descatha et al. (2012)	Y	Y	Y	Y	Y	Y	Y	Y
Palmer et al. (2014)	Y	Y	Y	N	Y	Y	N	Y
Broekstra et al. (2018)	Y	Y	Y	Y	Y	Y	Y	Y

Y=Yes, N=No, U=Unclear, NA=Not Applicable.
