# Supplementary material for: Incidence of Dupuytren’s disease following hand trauma: a systematic review
Source: J Hand Surg Eur Vol. 2025 Aug 1;51(1):6–13. doi: 10.1177/17531934251360545 (PMC12705875; doi:10.1177/17531934251360545)
Supplement: sj-pdf-4-jhs-10.1177_17531934251360545 - Supplemental material for Incidence of Dupuytren’s disease following hand trauma: a systematic review [file sj-pdf-4-jhs-10.1177_17531934251360545.pdf]

Online Table S2. Quality assessment of included studies using Newcastle-Ottawa Scale for cohort studies

Author & year	Selection				Comparability	Exposure		
	Representativeness of the exposed cohort	Selection of the non-exposed cohort	Ascertainment of exposure	Demonstration that outcome of interest was not present at start of study	Comparability of cohorts on the basis of the design or analysis	Assessment of outcome	Was follow-up long enough for outcomes to occur	Adequacy of follow up of cohorts
Bell (1977)	★		★					★
Stewart et al. (1985)	★		★		★	★	★	★
Kelly et al. (1992)	★		★	★		★	★	★
Livingstone et al. (1999)	★		★			★	★	
Abe et al. (2007)	★		★	★		★	★	★
Descatha et al. (2014)		★			★★		★	
Samulėnas et al. (2020)	★		★	★	★	★	★	★
van den Berge et al. (2021)	★	★	★	★	★★	★	★	★
Maasarani et al. (2022)	★	★	★	★	★★	★	★	
van den Berge et al. (2023)	★	★	★		★★	★		
Zimmerman et al. (2024)	★	★			★★	★	★	★

★ = one score
