# Supplementary material for: Incidence of Dupuytren’s disease following hand trauma: a systematic review
Source: J Hand Surg Eur Vol. 2025 Aug 1;51(1):6–13. doi: 10.1177/17531934251360545 (PMC12705875; doi:10.1177/17531934251360545)
Supplement: sj-pdf-5-jhs-10.1177_17531934251360545 - Supplemental material for Incidence of Dupuytren’s disease following hand trauma: a systematic review [file sj-pdf-5-jhs-10.1177_17531934251360545.pdf]

Online Table S3. Quality assessment of included studies using Newcastle-Ottawa Scale for case-control studies

	Selection				Comparability	Exposure		
Author & year	Is the case definition adequate?	Representativeness of the cases	Selection of Controls	Definition of Controls	Comparability of cases and controls on the basis of the design or analysis	Ascertainment of exposure	Same method of ascertainment for cases and controls	Non-Response rate
Haines et al. (2017)		★	★	★	★ ★	★	★	
